# Supplementary material for: Urogenital schistosomiasis among pre-school and school aged children in four districts of north western Tanzania after 15 years of mass drug administration: Geographical prevalence, risk factors and performance of haematuria reagent strips
Source: PLoS Negl Trop Dis. 2022 Oct 12;16(10):e0010834. doi: 10.1371/journal.pntd.0010834 (PMC9591047; doi:10.1371/journal.pntd.0010834)
Supplement: Attachment — Submitted filename: Response to reviewer comments.docx [file pntd.0010834.s001.docx]

**Response to reviewer comments**

**Dear Editor**

**Here are point to point response to reviewer comments**

| **S/no** | **Reviewer comments** | **Response to reviewer comments** |
| --- | --- | --- |
| **Reviewer 1** | | |
| 1 | The manuscript is primarily descriptive, with no particular hypothesis put forth or tested. The authors do address whether or not reagent strips are useful for adjunct diagnosis for S. haematobium infection, which would be informative for other programs, but did not lay out a clear comparison of how "usefulness" was tested or determined. | This was an analytical cross-sectional study aimed at describing the current status of S. haematobium, its related factors and assess the performance of urine reagent strip after multiple rounds of MDA. We have included corrections with a question and rationale   - In the data analysis section, we have explained how the useful/performance of the urine reagent strip was evaluated. |
| 2 | What was the relationship between the 86 schools surveyed in 2005/2006 and 88 schools surveyed in 2019? A great deal of overlap or were they mostly different schools. Rather than one map combining all the schools from the baseline (Figure 1) and then district level maps (Figs 3-6) for the follow up survey, it would be more useful to have side by side baseline and 2017 maps for each district so the changes can be more readily seen. As part of these paired maps, the number of rounds and intervals of MDA should be provided for each district. Figure 2 is not very useful at all and should be deleted. | - The only relationship is that the 86 schools and 88 were in the same districts for the study conducted in 2005/06 and 2019, but not necessarily the same school - The parasitology data for the schools included in the 2005/06, is not available for each school, only prevalence for each district - The map in figure 2 for 2017, was developed using the presence of red urine questionnaire, with not parasitological data. - We do agree to delete figure 2, is not useful - Since, 2006, school children in these districts have been receiving a single annual MDA up to 2017, however in 2018, there was no treatment for all the districts. |
| 3 | The authors stated that the surveyed schools were classified according to ecologic zone (distance from water body) but no analysis based on ecologic zone is presented. | - Considering the focal nature of schistosomiasis distribution, with schools located close to water bodies likely to have higher risk of SAC having the infection, only schools close to water bodies were included. This description has been added in the manuscript. - We acknowledge this is a limitation of the study |
| 4 | Similarly, the authors state that a questionnaire was used to collect demographic information, water source, knowledge of open water sources, symptoms, and participation in MDA; however, none of these data were analyzed or presented in the results. Only differences in prevalence according to districts were presented but there was no indication of why some districts were higher or lower than others. | **-**The questionnaire recorded only demographic information mainly age, sex, class, school name, district and region participation in mass drug administration. These were used to analyses the risk factors associated with the post-MDA *S. haematobium* infection.  - Additional information has been added in the discussion section to explain the observed difference in prevalence of *S. haematobium* between the study districts. |
| 5 | Results of reagent strips for microhaematuria were recorded semi-quantitatively but not reported or compared to eggs counts. One useful thing this paper could present is the relationship between infection intensity based on egg counts and semi-quantitative microhaematuria--is it the same at baseline and post-several rounds of MDA or does having treatment rounds change the dynamic? This information would make the paper more attractive to a wider audience. | - We agree with the reviewer on this point, however, re-reviewing our data set, the urine reagent strips were not recorded quantitatively. The data were qualitatively recorded as positive and negative (categorical). Correction has been done. - Note that, we did not have baseline data to do comparison for this study. We could not access the data of 2005/06 to do comparison. Only data on the prevalence of *S. haematobium* for each district were available. |
| 11 | The way Table 2 is laid out and labelled suggests that it is urine filtration that is being evaluated rather than the urine reagent strip. | - Thank you for the observation, correction is done. The correct test is Urine reagent strip |
| 12 | Figure 10 should be a bar graph rather than a line graph as there is no continuum between the districts like there would be if time were being plotted. Where are the 2005/2006 data from Itilima? Line 309 suggests 15 years of MDA but the figure legend states it is a decade. How many rounds of MDA were delivered in each district? | - Correction done on the figure, a bar graph has replaced the line graph - In the 2005/06 mapping, Itilima district was part of the Bariadi district, no separate data were available for this district - From 2006, all the districts have received a single annual round of MDA to 2017 |
| 13 | The authors state that some districts have hotspot areas but do not define what constitutes a hotspot or what might be different between hotspot and non-hotspot areas. | - Correction has been done to remove the word hotspot. This is more used in control programme with a predefine infection decline and areas which do not meet the expected reduction in prevalence are termed as persistent hotspot areas |
| 14 | As a purely descriptive study that there are more infections in some district than others and that prevalence differs by age and sex, the conclusions are supported by the data. However, the criteria that many rounds of MDA do not change reagent strip performance is not directly evaluated other than showing the sensitivity and specificity data. But whether sensitivity was higher pre-MDA is not addressed. | - This is very true and we agree with the reviewer. The lack of comparison data from the baseline mapping survey of 2005/06 has affected this evaluation - We acknowledge this and include as a limitation of the study |
| 15 | The English is good enough that the intentions of the paper are easily understood but it has numerous subject/verb agreement errors, missing articles, and simply oddly worded sentences that are not clear (e.g. lines 56-57, 102-106, 119, 121, that it would greatly benefit from copy editing.  line 93--urine filtration for eggs is the standard method for S. haematobium diagnosis but it is not a "gold" standard. line 202--mentions a third laboratory technician but it was never disclosed that 2 read each slide to begin with. Is that correct? line 257 says the majority of male patients had heavy infections but this is not true as only 19.5% did. However, the majority of heavy infections were in males is a true statement. There a simple error like this throughout the paper that should be corrected. | - We thank the reviewer for this observation. We have addressed all the corrections suggested by the reviewer |
| 16 | As presented, the study is primarily descriptive and not very generalizable to other settings. The frequency of MDA distribution is not clear (15 years in the title but a decade in line 25) and without a clear history for each district it is not possible to determine whether hotspots are the result of some ecological difference or simply reflect more, or more consistent, MDA is some areas compared to others. However, these same data could be used to develop a much more compelling manuscript. For example, as mentioned in the results section, do multiple rounds of MDA in a district change the relationship between egg count and level of microhaematuria? | - We have made changes to address all the corrections suggested by the reviewer. MDA in all the districts involved in the study started in 2000 and school children received a single round of MDA to 2017. Between this period different funding agencies supported the programme - In the discussion section, we have added a section to account for the observed variation in prevalence between the districts. - The baseline results of 2005/06 are not available, to be able to assess what the reviewer is suggesting. We agree, this would have added value to the paper. |
| **Reviewer 2** | | |
| 1 | Schistosomiasis still poses a huge public health concern in many sub-Saharan countries. Many of these countries including Tanzania have implemented mass drug administration (MDA) targeting primary school children for the control. After a decade of national MDA in Tanzania the strategy and infection diagnostic tools have been evaluated to inform the next stage of the schistosomiasis control activities. The findings from the study provide an updated geographical prevalence giving insight on the planning of MDA. The prevalence of S. haematobium infection have significantly declined informing about the success of the programme. Appropriate design has been used to evaluate the success of the control programme.  The results pointed to the success of the repeated rounds of mass drug administration. The urine reagent strips remain as a useful diagnostic tool for rapid monitoring of urogenital schistosomiasis in areas with moderate to high prevalence. The study set out clear objectives that have been addressed and the also the study got thorough ethical approval before implementation. This undertaking permitted to gauge the success of the MDA programme and also assisted mapping the distribution after several rounds of MDA. The way the data have been presented provides an exceptional example to be applied in different settings and map the way forward on the control exercise. | - We thank the reviewer for this observation |
| 2 | The results are outlined appropriately and following the objectives set out to answer. The figures are clear and well presented with the exception of Figure q10 that would be more appropriate presented as a bar graph instead of lines. The lines do not represent the linkage between the different points. The data availability provides the opportunity to create big data set and reveal the trends combining with other countries in sub-Sahara. | - We thank the reviewer for this observation - Figure 10 has been changed as per reviewer |
| 3 | The conclusions are well supported by the data and the presentation is clear and showing the trends. the authors mentioned the study limitations while the analysis makes it clear on the current trends in which mass treatment is the mainstay of control initiatives in the sub-Sahara region. The comparison to the original surveys before the MDA initiation is important and showing the progress made so far after several rounds of MDA. The data and analysis give an opportunity to evaluate the progress and see the gaps in the control initiatives. | - We thank the reviewer for this observation. We acknowledge that we could not get the parasitological data of the survey which was conducted in 2005/06, which would have allowed us to do comparison before and after. Only the prevalence data calculated from urine filtration test are available with no data of the eggs counts |
| 4 | The data is professionally handled and given in a clear manner. However, I noticed the mode of presentation of figure10 may require revision to show as a bar graph instead of lines joining different study sites. The same data can be appropriately presented as bars. | - Correction has been done for Figure 10. The figure is presented in bars. However, during the survey of 2005/2006, Itilima district was part of the Bariadi district and currently is an independent district. Data for the baseline are part of the Bariadi district and not included in Itilima district |
| **5** | There general minor corrections need to be observed at the following page points: -page 27-A cross sectional study -page 116 form to take to their parents -page 119 are kept in closed cabinet -page 121 were treated using PZQ -page 141 remove man-made inland and natural -page 198 please check the pore size if not in micro metres -page 277 line should be linked to line 278 -page 389 its performance -page 434 this study would not be .... -Change figure10 lines to stand alone bars | - We thank the reviewer for all the observations and we have worked on all the comments |
| **Reviewer 3** | | |
| 1 | Line 327: Is the 21.7% mentioned here, correct? Should it be 20.7%? | This is 20.7%, not 21.7%, correction done |
| 2 | There are a few minor language corrections, e.g.: line 27: “Cross-sectional study” should be “A cross-sectional study” •       Line 38: “Low” should be “Light” •       Line 88: “majority” should be “the majority” •       Line 101: “needs to re-assessment” should be “needs re-assessment” •       Line 102: “ad” should be “and” •       Line 141: “inland” is repeated •       Line 256: “Majority” should be “More”. •       Line 270: “were likely” should be “were more likely” | - All the proposed correction have been addressed |
| 3 | This is a really rich sample of over 20,000 children from four districts, with 250 children from each school. This area has been under treatment for a long time, and so it is interesting to see the level of infection in this area, and any change from baseline. Given the size of the sample size, I think there are a number of additional useful analysis that could be carried out. In particular looking at the distribution of infection by sub-district, and looking at school-level hotspots of infection. | - We do agree with the reviewer, at present, there is an on-going effort to map the disease prevalence at sub-district levels rather than the district level. In Tanzania, this is a ward level. - The current data set have data which were recorded at district levels and are presented at district level. - However, after this study more work is required at sub-district levels that identifies the hotspot areas for the national programme to consider for continued mass treatment. |
| 4 | Given the sample size, and the WHO push for sub-district level implementation, is it possible to define results (and maps) by sub-district? That would help to fine-tune the implementation approach | - This is good suggestion; however, we did not record the wards’ location for each school, which will allow sub-district data analysis |
| 5 | I didn’t see any analysis of the difference in infection markers between the three ecological zones (distance from lake). Can this be added? | - Considering the focal nature of schistosomiasis distribution with the community living close to water bodies having the highest risk, we only considered schools which were close to water sources, in the first ecological zone. We have clarified this in the manuscript |
| 6 | The authors talk about many hot-spots at the school-level remaining. How many of these falls into that category? | - I have included all the schools which had prevalence above 10% for each district. - Note that we have avoided the word hotspot areas, we did not provided definition for this and the word fits more in areas where intervention has been well monitored and definition provided |
| 7 | I would say there is only a modest reduction in prevalence from baseline, which in a sense is disappointing. However, macrohaematuria is very low which is reassuring. As a more direct measure of morbidity, it is more reassuring to see this fall than prevalence. | - We do agree with the reviewer |
| 8 | How many of the districts / sub-districts have reached the metric of <1% heavy infections which indicates elimination as a public health problem? | - We have shown the distribution of light and heavy infection in each district, unfortunately, none of the district included in the study had reached a metric of <1% heavy infection. All are above 10% |
| 9 | Given that there have been so many rounds of treatment in this area, are there conclusions that can be drawn about the usefulness of the MDA approach? E.g. that it can be used to suppress infection but cannot take us to elimination. | - Honestly, without integrating other intervention measures such as improved water supply, sanitation and hygiene and strengthening the primary health system, it will be difficult to reach elimination stage. No question that MDA approach is useful for suppressing infection and morbidity control but cannot take us to elimination stage |
| 10 | Check author affiliations. Is Cecilia Uisso affiliated to RTI? | - Correction done |
| 11 | It is a long time since baseline. Were any intensity measures available from baseline? Have there been any other surveys between the two time-points? | - Unfortunately, we made follow-up with the group which conducted the baseline survey in 2005/06, we managed to get only the prevalence (number infected/number screened) data for each district. - No other survey(s) were conducted in between and after the 2005/06 surveys. The one presented here is the second survey. |
| 12 | Aims: “Thus, the overall aims of the present study were to (i) determine the prevalence ad intensity of infection of S. haematobium, prior to implementation of mass preventive chemotherapy to allow monitoring of the impact of treatment on infection prevalence and intensities”. Consider re-wording, it’s not prior to implementation. | - Corrections done |
| 13 | Line 124: you say that these areas are highly endemic for SCH, but pre-MDA mapping categorized them as low to moderate endemicity. Which is correct? | - Corrections done- low to moderate endemicity |
| 14 | Lines 208-209: “Generated maps categorized prevalence levels based on WHO categories (0%, 0.1-10.0%, 10.1-20.0%,>20%)” Are they the cut-offs? I think for SCH, they are 0%, 10%, and 50% | - This is true, changes are made. However, we believe that 0%, 0.1-10%, 11-50% and above 50%, these are the categories ranges |
| 15 | Many fewer children were sampled in Shinyanga district – why is that? | - In this district there are few schools |
| 16 | Lines 252 – 254. If overall intensity was 15.78 e/10ml, I don’t understand how males can be 3.26 e/10ml and females 1.68 e/10ml. I think for female instead of “1.68 +/- 13.29” it should be “13.29 +/- 1.68” | - Correction has been done, using the geometrical mean, Male=17.3eggs/10mls of urine and Female=13.8eggs/10mls of urine |
| 17 | Figure 9 – in multivariate analysis, was there no longer an association between haematuria and being infected with *S. haematobium*? That would be an interesting finding. | - We have reviewed the data analysis and clearly there is an association between *S. haematobium* and microhaematuria |
| 18 | Figure 10: Recommend redrawing as bar chart, rather than line chart. | - Correction has been done |
| 19 | Titles for Figures 3-6 need more information. Are these the results from the current study? | - Correction has been done; more details added |
